# Successful Low-Dose tPA for Mechanical Mitral Valve Thrombosis in a Patient with Postinfarction Contained Left Ventricular Free Wall Rupture

**DOI:** 10.1155/2021/5532728

**Published:** 2021-07-17

**Authors:** James Livesay, Emmanuel Isang, Hassan Tahir, Raj Baljepally

**Affiliations:** ^1^Department of Medicine, University of Tennessee Graduate School of Medicine, Knoxville, TN, USA; ^2^Heart Lung Vascular Institute, University of Tennessee Medical Center, Knoxville, TN, USA

## Abstract

Prosthetic valve thrombosis is a potentially life-threatening complication diagnosed by a combination of clinical features and imaging modalities, but the optimal management in high bleeding risk patients remains controversial. Current treatment options for prosthetic valve thrombosis included surgery, thrombolytic therapy, and anticoagulation. We present a very unusual case of a patient with a recent ST-elevation myocardial infarction complicated by contained left ventricle free wall rupture and mechanical mitral valve thrombosis. Deemed a high surgical risk candidate, low-dose tissue plasminogen activator was used despite significant bleeding risk from contained left ventricle free wall rupture, which resulted in resolution of the thrombus. To the best of our knowledge, this is the first report of successful thrombolytic therapy for prosthetic mechanical mitral valve thrombosis in a patient with recent postmyocardial infarction contained left ventricular free wall rupture.

## 1. Introduction

The most common complications after native valve replacement include prosthetic valve thrombosis (PVT), pannus formation, embolic events, bleeding, and infective endocarditis [1]. The rate of PVT is higher in patients with mechanical valves versus those with bioprosthetic valves, with an annual rate up to 5.7% in the mechanical valve group [2]. Mitral mechanical PVT is twice as common as aortic mechanical PVT, and rates of thromboembolism are higher for certain types of mechanical valves [3]. Any patient with a known mechanical valve that presents with clinical signs of valve obstruction such as a new murmur, subdued clicks, symptoms or signs of heart failure, thromboembolic events, or elevated transvalvular gradients should be evaluated for PVT. Prosthetic valve mean gradients increased by 50% from the baseline in the setting of acute presenting symptoms, and subtherapeutic anticoagulation is consistent with PVT [4]. The current management strategies for PVT include surgical intervention, thrombolytic therapy, and anticoagulation [2]. However, the best treatment option depends on many factors, including right versus left-sided valve involvement, thrombus burden, surgical risk, and patient preference.

## 2. Case Presentation

A 52-year-old woman with a history of chronic kidney disease status post kidney transplant, remote history of mitral valve endocarditis status post St Jude mechanical mitral valve replacement, and heart failure presented with an ST-elevation myocardial infarction. She underwent emergent left heart catheterization revealing a 100% occluded obtuse marginal branch for which percutaneous intervention with stent implantation was performed. Follow-up transthoracic echocardiogram (TTE) showed a low normal ejection fraction of 52% and normal mitral valve gradients. She was discharged home on guideline-directed medical therapy postacute myocardial infarction.

Approximately three weeks later, she presented with acute dyspnea, orthopnea, and acute hypoxic respiratory failure. Her international normalized ratio (INR) on presentation was supratherapeutic at 8.29 with a repeat the following day of 2.02. She was initially treated for a heart failure exacerbation with bilevel positive airway pressure and aggressive intravenous diuretics. A TTE was repeated given her acute clinical change, revealing a severely reduced ejection fraction of 30-35%, abnormal prosthetic mitral valve gradients, and restricted posterior valve leaflet mobility. The mitral valve max pressure gradient was 21.1 mmHg with a mean gradient across the mitral valve of 11.0 mmHg. The TTE also revealed a tricuspid regurgitation (TR) max velocity of 343.9 cm/sec, TR max PG 47.3 mmHg, RVSP 50.3 mmHg, and pressure half-time of 2.5 cm^2^. PVT was diagnosed based on her acute clinical symptoms and echocardiography findings.

**Figure 1:**
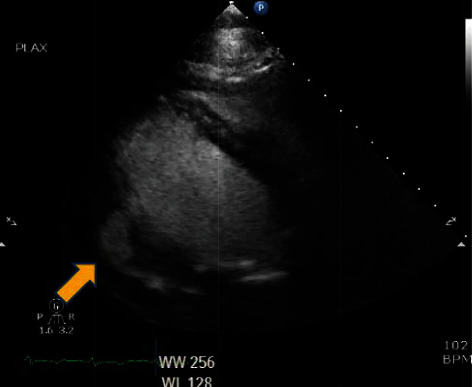
Parasternal long axis view of contrast leaking into the pericardial space on echocardiogram.

**Figure 2:**
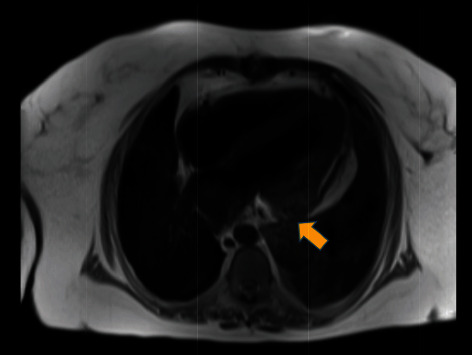
Cardiac magnetic resonance view of the contained left ventricle free wall rupture.

Incidentally, contrast TTE also revealed a posterolateral pseudoaneurysm with contrast extending into the pericardial space concerning for left ventricular free wall rupture (Figure 1). A transesophageal echocardiogram (TEE) was considered, but the patient required high levels of supplemental oxygen and therefore deemed high risk by anesthesia. Given her history of renal transplant and worsening renal function, cardiac magnetic resonance was performed instead of a CT angiogram which confirmed a contained left ventricle free wall rupture (Figure 2).

**Figure 3:**
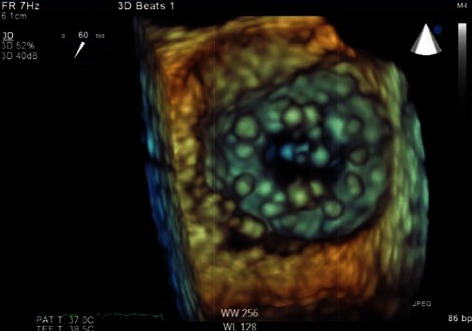
3D transesophageal echocardiogram of the normally functioning mechanical mitral valve at 60 degrees following treatment with tPA.

**Figure 4:**
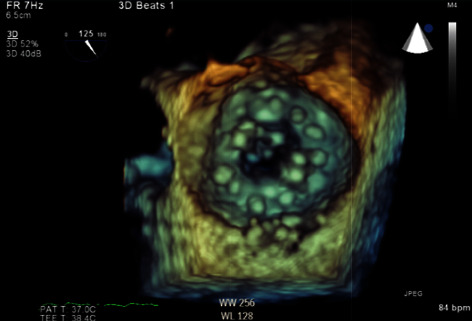
3D transesophageal echocardiogram of the normally functioning mechanical mitral valve at 125 degrees following treatment with tPA.

Due to mechanical mitral PVT and left ventricular pseudoaneurysm, felt to be secondary to recent myocardial infarction, cardiothoracic surgery was consulted. She was considered inoperable due to multiple factors: recent myocardial infarction on dual antiplatelet therapy, heart failure with reduced ejection fraction, acute respiratory failure, worsening renal function, prior renal transplant, and prior open-heart surgery. Management options were extremely limited, but after discussion with the patient's family, low-dose tissue plasminogen activator (tPA) was administered and infused at 25 mg over six hours. The following day, patient's dyspnea improved. She underwent a TEE showing a normally functioning mechanical mitral valve with a mean gradient across the valve of 5 mmHg. There was no evidence of thrombus and the left ventricle posterior wall pseudoaneurysm was unchanged (Figures 3 and 4).

Although her initial hospital course was complicated by renal failure requiring continuous renal replacement therapy, prolonged respiratory failure requiring high doses of supplemental oxygen, and hypotension limiting guideline-directed medical therapy, she recovered gradually with conservative management of the contained posterior wall rupture. She continued to show improvement and was discharged home following a twenty-five-day hospitalization. She was seen in the ambulatory setting two months later without complaints of dyspnea or orthopnea and an echocardiogram showing a mean mitral valve gradient of 8.9 mmHg with no pericardial effusion. Since then, she has been seen multiple times in our outpatient clinic and is doing very well.

## 3. Discussion

The current prevalence of mitral and aortic valve disease in the United States is approximately 2.5% in the general population and up to 10% in people 75 years or older [2]. Treatment of valvular disease includes surgical replacement such as mechanical and biological valves and, more recently, transcatheter technology. While mechanical valves are more durable, they have a significantly elevated risk of developing thrombus with symptomatic obstructive mechanical valve thrombosis occurrence ranging from 0.3% to 1.3% per year [1]. Management of PVT remains challenging and depends on several patient factors: thrombus location, thrombus size, the patient's functional status, and surgical and bleeding risk [5].

PVT should be considered when patients present with acute symptoms, new murmur, signs of heart failure, diminished valve sounds, and thromboembolic events [1]. The exact incidence of PVT is likely underestimated as appropriate imaging is either not performed or suboptimal. TTE, TEE, fluoroscopy, and CT imaging are used to assist in diagnosing PVT [2, 4]. Thrombolytic therapy for right-sided PVT is considered if clot persists despite intravenous heparin. However, the management of left-sided PVT is more problematic. Factors that favor surgery include the availability of surgical expertise, low surgical risk, recurrent thrombus, NYHA class IV heart failure, possible pannus, and coronary artery disease requiring revascularization. While factors that favor thrombolytics include lack of available surgical expertise, high surgical risk, first-time thrombosis, NYHA class ≤ III heart failure, no coronary artery disease, and no pannus formation. [2, 4]. Other advantages of thrombolytic therapy over surgery include its ubiquitous availability and ease of use. Intravenous heparin is recommended in each case with thrombolytic therapy if valve thrombus persists [2].

Kapos and colleagues presented a case of a 52-year-old female presenting with signs of cardiac congestion. An urgent TTE revealed an elevated diastolic transmitral gradient of 21 mmHg with impaired movement of one of the disc. TEE confirmed the TTE findings, but no mass was seen. Given her symptoms and echocardiographic findings, she was diagnosed with a PVT and started on unfractionated heparin. Despite five days of therapy, the mitral stenosis remained unchanged, and she was started on low-dose-ultraslow fibrinolytic therapy [1].

Kalҫik and colleagues present a case of a 57-year-old female that presented with NYHA class III dyspnea. TTE revealed an increased mean mitral transvalvular gradient of 15 mmHg with a decreased mitral valve area of 0.9 cm^2^. She had no contraindications to thrombolytic therapy and was started on low-dose tPA 25 mg over the course of 25 hours (low-dose and ultraslow fibrinolytic therapy). Repeat TTE showed a decreased transvalvular gradient and relaxation in the restricted leaflet. She was discharged on oral anticoagulation and did not require surgical intervention [6].

The ultraslow PROMETEE trial evaluated the use of low-dose slow infusion of tPA (25 mg over 6 hours) in patients with mechanical PVT. Thrombolytic therapy was administered to 114 patients under TTE guidance. They found a low-dose ultraslow infusion of tPA was associated with an overall success rate of 90% and low morbidity and mortality in each NYHA class except class IV [7]. Low-dose thrombolytic therapy is a viable alternative to surgical intervention in patients with PVT but not well studied in patients at an elevated bleeding risk. Although an ultraslow tPA infusion (25 hours) is probably more appropriate in patients with higher bleeding risk, it is not an effective option in unstable patients such as ours, who require more rapid treatment. Overall, however, the benefit of thrombolytic therapy for PVT may outweigh the risk in critically ill inoperable patients, despite the elevated bleeding risk.

## 4. Conclusion

The optimal management option is unclear in patients presenting with both PVT and extremely high bleeding risk. Our patient is unique in that she presented with both acute respiratory failures from mechanical mitral PVT and a left ventricular free wall contained rupture after a recent ST-elevation myocardial infarction. A successful outcome in our highly unusual case illustrates that despite the extreme bleeding risk, low-dose thrombolytics are potentially lifesaving and should always be considered, especially in inoperable patients.
